# Short-Term Outcomes of Visual-Aid-Based Motivation on Children’s Oral Hygiene: A Randomized Controlled Trial

**DOI:** 10.3390/children13010109

**Published:** 2026-01-12

**Authors:** Merve Candan, Melike İdacı, Alper Çamgöz, Hatice Hatipoğlu, İmran Gökçen Yılmaz Karaman

**Affiliations:** 1 Department of Pediatric Dentistry, Faculty of Dentistry, Eskisehir Osmangazi University, 26040 Eskisehir, Türkiye; 2 Department of Pediatric Dentistry, Faculty of Dentistry, Afyonkarahisar Health Sciences University, 03030 Afyonkarahisar, Türkiye; 3 Department of Psychiatry, Faculty of Medicine, Eskişehir Osmangazi University, 26040 Eskişehir, Türkiye

**Keywords:** dental plaque index, halitosis, health education, dental, motivation, oral hygiene, pediatric dentistry

## Abstract

Background and Objectives: The aim of this study is to evaluate the effect of positive and negative visual aids used during verbal–active oral hygiene education on oral hygiene-related behaviors in children aged 7 to 14 years. Materials and Methods: In this single-blind design, sixty children were randomly assigned to three groups: G1:Positive visual aid, G2:Negative visual aid, and G3:Verbal–active education. Oral hygiene was evaluated using the Silness–Löe Index (plaque) and Rosenberg Organoleptic Scale (halitosis) at baseline, one week, and one month. Measurements were taken at baseline, at the end of the first week, and at the end of the first month. Data were analyzed using split-plot ANOVA. Results: The test groups did not show any statistically significant differences in terms of age (F = 0.530, *p* = 0.449) or gender (χ^2^ = 1.600, *p* = 0.449). Additionally, the groups were similar in terms of clinical variables, including dentition stage (permanent or mixed) (χ^2^ = 5.566, *p* = 0.062), presence of malocclusion (χ^2^ = 3.801, *p* = 0.150), and presence of anterior dental caries (χ^2^ = 1.250, *p* = 0.535). Significant reductions in both plaque and halitosis scores were observed over time in all groups (*p* < 0.001), and there were no statistically significant differences between the types of intervention (*p* > 0.05). Conclusions: This study demonstrated that both verbal education aided by positive and negative visuals and structured-only verbal education improved children’s oral hygiene and halitosis scores in the short term.

## 1. Introduction

Oral and dental health plays a fundamental role in terms of children’s physical development, nutrition, and psychosocial well-being. The deterioration of oral health adversely affects not only local problems but also systemic health and children’s oral health-related quality of life [1,2,3]. Therefore, preventing oral diseases before they occur and instilling correct hygiene habits in children at an early age is a primary objective of preventive dentistry [4].

Traditional Oral Hygiene Education (OHE) applied to ensure plaque control in children is generally based on verbal information transfer. However, Piaget’s theory of cognitive development argues that children’s learning skills vary according to their developmental stages and that verbal explanations requiring abstract thinking may be insufficient in providing permanent learning, especially in children during the concrete operational stage. In this developmental process, learning becomes much more effective through interaction with concrete objects and direct experiences [5]. Consequently, supporting OHE protocols with visual materials is of critical importance in terms of internalizing theoretical knowledge by concretizing it.

The content of visual materials is the main factor determining motivational processes. In the psychology literature, two basic motivational mechanisms are defined for behavior change: “approach motivation,” based on the individual’s desire to reach a reward or a desired state (e.g., having white and healthy teeth), and “avoidance motivation,” based on the desire to avoid a negative outcome (e.g., fear of caries, pain, or tooth loss). Some studies on general health behaviors have shown that these types of motivation can be effective on health-related decision-making processes and habit formation [6,7,8]. In the context of oral health, in the limited number of studies examining the motivational effects of healthy and decayed tooth visuals in children, evaluations have predominantly been conducted at the levels of attitude, perception, and behavioral intention [9]. However, the impact of these stimuli in children may differ from adults due to developmental and neurobiological factors. Neurophysiological studies suggest that the pediatric brain does not prioritize the processing of negative stimuli to the same extent as adults [10,11].

Although the effects of positive and negative visuals on children’s attitudes have been examined in the literature, no randomized controlled study comparing the direct effects of these tools on oral hygiene and clinical parameters has been found. Current research mostly focuses on parents’ perceptions; the relationships between children’s individual behaviors, responses to education, clinical indicators, and the types of visual stimuli used have not been sufficiently elucidated [12,13].

In this direction, the aim of the present study is to comparatively examine the effects of verbal instruction, positive visual-aided instruction, and negative visual-aided instruction methods on children’s oral hygiene habits and clinical plaque scores. The null hypothesis of the research is that there is no statistically significant difference among the different education models applied in terms of children’s plaque scores and hygiene habits. In this context the alternative hypothesis predicts that visual-aided education models provide more effective plaque control compared to verbal education and that the quality of the visual content used (positive or negative) creates a significant difference in clinical outcomes. Since visual materials are more compatible with the learning characteristics of children in the concrete operational stage, it was hypothesized that these groups would exhibit significantly lower plaque scores compared to the verbal-only group.

## 2. Materials and Methods

### 2.1. Sample Selection

This study was conducted in accordance with the Declaration of Helsinki and approved by the Ethics Committee of Eskişehir Osmangazi University (Approval Date/Number: 5 June 2024/36). This clinical trial was also designed and conducted in accordance with the CONSORT (Consolidated Standards of Reporting Trials) statements. Clinical Trial Number: NCT07150429, ‘Impact of Visual Stimuli on Pediatric Oral Hygiene Performance’, Registration Date: 25 August 2025/Eskişehir/Türkiye, Link: https://clinicaltrials.gov/study/NCT07150429. The required sample size was determined using an a priori power analysis (G*Power version 3.1.9.7; Heinrich Heine University, Düsseldorf, Germany). For the Split-plot ANOVA (within-between interaction) model, the calculation was based on a medium effect size (f = 0.25), a conventional significance level (alpha) of 0.05, and a high power (1-beta) of 0.95. The analysis indicated that a minimum of 54 participants was required to achieve statistical robustness. To account for potential drop-outs during the follow-up period, 60 children aged 7–14 years who presented to the Department of Pediatric Dentistry at the Faculty of Dentistry, Eskişehir Osmangazi University, were included (20 children per group). Informed consent was obtained from both the parents and the children, and participants were assigned at random into the three study groups.

The randomization process was carried out using a reliable online randomization tool (www.researchrandomizer.com). To ensure allocation concealment, the randomization process was conducted by an external researcher (M.C.) before the intervention phase of the study commenced. The group assignment list was kept inaccessible to both the educator and the evaluator until all baseline measurements of the respective participant were completed (until baseline scores were recorded). All 60 participants who were randomized at the beginning of the study completed the entire follow-up period, and no data was lost.

The inclusion criteria for the present study were: (1) children who had not previously received OHE, (2) those with carious lesions scored no higher than 4 on the ICDAS scale, and (3) systemically healthy children between the ages of 7 and 14. The study population consisted of male children, as well as female children who had not yet reached menarche. Conversely, the present study excluded children who declined to participate, girls who had reached menarche, children with systemic disorders, and those presenting with intraoral conditions such as defective restorations that could facilitate plaque retention. This was implemented due to the knowledge that improper restorations and ICDAS 5 or 6 deep, cavitated lesions directly contribute to bacterial accumulation and thus affect clinical plaque scores, independent of oral hygiene motivation [14,15]. Since the primary focus of the study is behavioral motivation, this limitation was deemed necessary to isolate the confounding effects of these severe clinical factors and to confirm that the effect originated from behavior change. Furthermore, the reason for excluding female participants who had entered menarche from the sample was to control for the potential confounding effects of hormonal fluctuations associated with the menstrual cycle on gingival health and their possible impact on the plaque score [16].

### 2.2. Study Design

All children included in the present study underwent a dental examination, during which their age, gender, dental plaque scores according to the Silness and Löe Plaque Index [17], and halitosis scores based on Rosenberg’s organoleptic scale [18] were recorded. Dental plaque scores were assessed separately for the anterior and posterior regions of the dentition, as well as for the overall dentition (total plaque score). The anterior dental plaque score represents the total dental plaque score of the anterior teeth, including the canines, while the posterior dental plaque score includes the total dental plaque score of the teeth located distal to the canines. The total dental plaque score represents the mean plaque accumulation across all teeth present in the oral cavity. The primary outcome was the change in total plaque and halitosis scores over time. Secondary outcomes included anterior and posterior plaque scores.

Participants were instructed to bring their current toothbrushes and toothpaste to the initial session, where these items were evaluated. The educational intervention across all groups was conducted within a structured protocol lasting an average of 10 min. The education incorporated verbal–active communication methods and was supported by a practical demonstration of both brushing techniques and the use of dental floss on a dental model. Brushing and flossing were explained using appropriate techniques widely accepted in the literature [19,20]. Except for the content of the visual materials, the content, duration, and presentation format of the education were standardized across all groups.

Sixty children participated in the present study and were randomly assigned to three groups. The initial group represented the positive visual group (*n* = 20), wherein, alongside verbal and active education, participants were shown with a representative visual illustrating periodontally healthy, well-aligned, and caries-free teeth as the anticipated result of consistent toothbrushing. The second group represented the negative visual group (*n* = 20). Alongside the verbal and active education, this group was shown a representative visual of decayed teeth, periodontal problems, and malalignment to illustrate the possible consequences of neglecting oral hygiene. No visual materials were presented to the control group.

**Figure 1:**
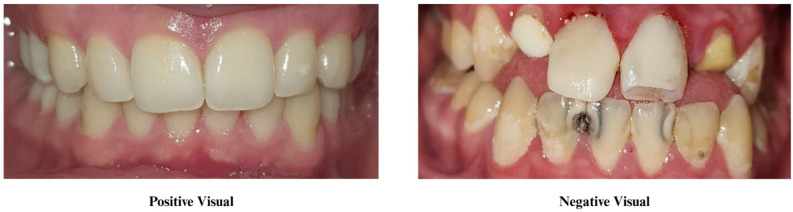
The representative visuals used in the study.

In the visual-aided groups, participants were shown high-resolution A4-sized (21 × 29.7 cm) printed materials corresponding to their respective motivation type (positive or negative) at the conclusion of the education. These images, depicting positive and negative outcome scenarios, are provided in Figure 1. Written informed consent was obtained from both the children and their legal guardians for the inclusion of patient photographs in Figure 1. The third group served as the control groups (*n* = 20) and received only verbal and active OHE. During the educational sessions, all groups were recommended to use similar oral hygiene products: manual toothbrushes with straight nylon bristles, dental floss, and 1 g of toothpaste containing 1450 ppm fluoride. The study was designed as a single-blind, assessor-blinded randomized controlled trial. Due to the nature of the educational intervention, the researcher delivering the OHE (M.İ.) could not be blinded; however, the researcher responsible for all clinical assessments, including plaque and halitosis scoring (A.Ç.), was blinded to group allocation.

### 2.3. Evaluation Parameters

All verbal and active OHE sessions were performed in a secluded environment within the patient waiting area, devoid of auditory and visual distractions. The educational sessions were delivered by M.İ. according to a pre-established standardized protocol. In the positive and negative visual groups, each participant was shown a single representative visual—either positive or negative—corresponding to their study group. A.Ç. performed the assessment of cases and recorded findings at baseline and throughout follow-up sessions.

**Figure 2:**
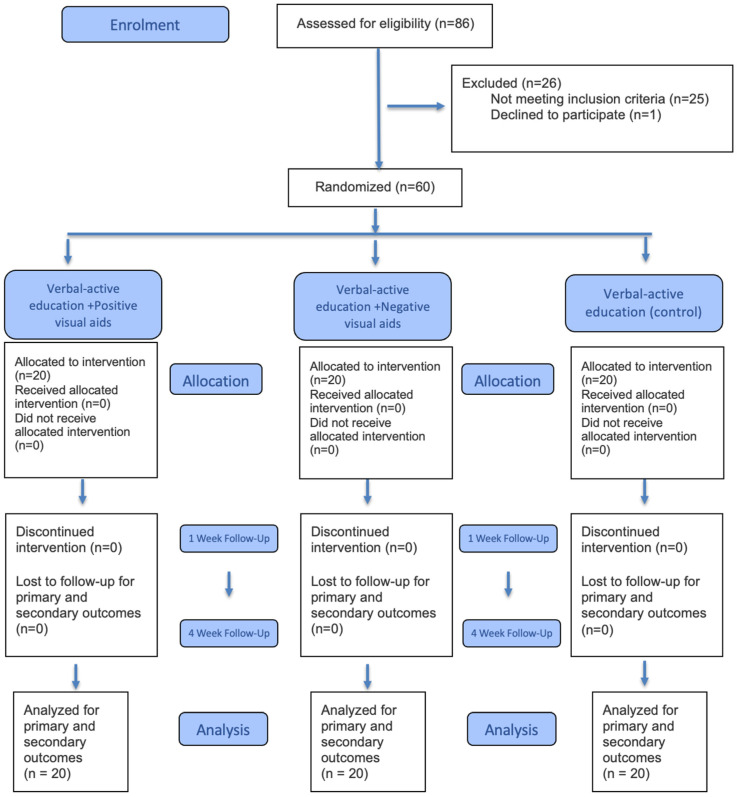
Consolidated Standards of Reporting Trials (CONSORT) flow diagram.

Following the completion of the OHE sessions, participants were evaluated using the Silness and Löe Plaque Index [17] and Rosenberg’s Halitosis Scale [18]. The Silness and Löe Plaque Index was modified to assess the presence and thickness of plaque on five surfaces of each tooth: mesial, distal, buccal, lingual, and occlusal. The Silness and Löe Plaque index was assessed using a 0–3 scoring system, ranging from no plaque (0) to an abundance of soft matter within the gingival pocket and/or on the tooth and gingival margin (3). Occlusal plaque was scored on a scale from 0 (no plaque) to 3 (heavy plaque accumulation). For each participant, the mean plaque index score was calculated as the sum of all tooth surface scores divided by the number of surfaces examined. The evaluated areas included the occlusal surface of the molar teeth to better reflect the participants’ overall brushing performance. Plaque scores were also assessed separately for the anterior segment, posterior segment, and as a total plaque score. Rosenberg’s Halitosis Scale [18], an organoleptic assessment method, was used to rate mouth odor on a scale from 0 (no odor) to 5 (extremely bad odor). All participants were invited for follow-up visits at one week and one month after the OHE session for data collection. All evaluations were recorded at three time points: the initial examination, the first week, and the first month. The study flowchart is presented in Figure 2.

The time points for follow-up visits were determined to evaluate the change in oral hygiene behavior in two stages: the short-term motivational effect and the transition toward habit formation. The 1-week follow-up was selected to observe the extrinsic motivation formed immediately after the intervention. This period also allowed for the assessment of the education’s acute effects. The 1-month follow-up aimed to evaluate the relatively long-term sustainability of the behavior. Furthermore, it examined the progression toward internalization, which signifies the onset of habit acquisition in the behavioral sciences literature [21,22].

### 2.4. Statistical Analysis

The statistical analysis of the data was conducted using IBM SPSS (Statistical Package for the Social Sciences) Version 26 software. Z-scores were calculated to detect outliers, and no outliers were identified. The normality of the distribution for continuous variables was assessed using skewness and kurtosis values, as well as boxplots and histograms. The skewness and kurtosis values were found to be within the range of −1.5 to +1.5, indicating that the data were normally distributed. In descriptive statistics, categorical variables were presented as frequencies and percentages, while continuous variables were expressed as means and standard deviations. The chi-square test was used to evaluate whether categorical variables such as gender and presence of anterior tooth decay were similarly distributed across groups. One-way ANOVA was employed to assess whether there were any differences in baseline measurements between the groups. To compare the effectiveness of intervention methods, changes observed in Silness and Löe’s Dental Plaque Index and Rosenberg’s Organoleptic Scale were analyzed using split-plot ANOVA. A *p*-value of <0.05 was considered statistically significant.

## 3. Results

Sixty participants were involved in the present study, comprising 30 boys and 30 girls. The mean age of the participants was 119.48 ± 21.76 months. The clinical characteristics of the participants are presented in Table 1.

The 60 participants were randomized into three groups of 20 each. No participants were lost to follow-up, and all randomized participants were included in the final analysis. These groups did not show any statistically significant differences in terms of age (F = 0.530, *p* = 0.591) or gender (χ^2^ = 1.600, *p* = 0.449). Additionally, the groups were similar in terms of clinical variables, including dentition stage (permanent or mixed) (χ^2^ = 5.566, *p* = 0.062), presence of malocclusion (χ^2^ = 3.801, *p* = 0.150), and presence of anterior tooth decay (χ^2^ = 1.250, *p* = 0.535).

The baseline measurements of the groups were compared. They showed no significant differences in terms of halitosis scores (F = 0.644, *p* = 0.529), total plaque scores (F = 1.088, *p* = 0.344), anterior plaque scores (F = 0.634, *p* = 0.534), and posterior plaque scores (F = 0.859, *p* = 0.429).

### 3.1. Analysis of Changes in Halitosis Scores: Between-Group and Within-Group Comparisons

The mean and standard deviation values for halitosis scores across the three measurements in each group are presented in Table 2. 

A split-plot ANOVA was conducted to assess the effect of the interventions on halitosis scores. There was no significant interaction between intervention type and time (Wilks’ Lambda = 0.930, F(4,112) = 1.028, *p* = 0.396, partial eta squared = 0.035). However, the main effect of time was large and statistically significant (Wilks’ Lambda = 0.655, F(2,56) = 14.766, *p* < 0.001, partial eta squared = 0.345). Halitosis scores decreased over time (Table 2). The main effect of the intervention type was not statistically significant (F(2,57) = 1.926, *p* = 0.155). In conclusion, all interventions led to a reduction in halitosis scores.

### 3.2. Analysis of Changes in Plaque Scores: Between-Group and Within-Group Comparisons

The mean and standard deviation values for plaque scores across different time points in the three test groups are presented in Table 3.

### 3.3. Analysis of Changes in Total Plaque Scores

The effect of the interventions on total dental plaque scores was examined. No significant interaction was found between intervention type and time (Wilks’ Lambda = 0.870, F(4,112) = 2.021, *p* = 0.096, partial eta squared = 0.067). However, the main effect of time was large and statistically significant (Wilks’ Lambda = 0.446, F(2,56) = 34.789, *p* < 0.001, partial eta squared = 0.554). Total dental plaque scores decreased over time (see Table 3). The main effect of the intervention type was not significant (F(2,57) = 0.031, *p* = 0.969). All interventions resulted in a reduction in total dental plaque scores.

### 3.4. Analysis of Changes in Anterior Plaque Scores

Changes in anterior dental plaque scores over time are shown in Table 3. The effect of the interventions on anterior dental plaque scores was examined using split-plot ANOVA. No significant interaction was found between intervention type and time (Wilks’ Lambda = 0.870, F(4,112) = 2.018, *p* = 0.097, partial eta squared = 0.067). The main effect of time was large and statistically significant (Wilks’ Lambda = 0.352, F(2,56) = 51.623, *p* < 0.001, partial eta squared = 0.648). Anterior dental plaque scores decreased over time (see Table 3). The main effect of intervention type was not significant (F(2,57) = 0.218, *p* = 0.805). All interventions resulted in a reduction in anterior dental plaque scores.

### 3.5. Analysis of Changes in Posterior Plaque Scores

Changes in posterior dental plaque scores over time are shown in Table 3. A split-plot ANOVA was conducted to evaluate the effect of the interventions on posterior dental plaque scores. No significant interaction was observed between intervention type and time (Wilks’ Lambda = 0.921, F(4,112) = 1.173, *p* = 0.327, partial eta squared = 0.040). The main effect of time was large and statistically significant (Wilks’ Lambda = 0.750, F(2,56) = 9.352, *p* < 0.001, partial eta squared = 0.250). Posterior dental plaque scores decreased over time (see Table 3). The main effect of the intervention type was not statistically significant (F(2,57) = 0.125, *p* = 0.882). All interventions resulted in a reduction in posterior dental plaque scores.

## 4. Discussion

According to data from the WHO, oral diseases are the most common diseases, affecting nearly half of the global population. They can impact individuals of all ages, from infancy to old age. Although dental caries is a preventable condition, the global prevalence of caries in primary teeth is estimated to be around 43% [23]. For this reason, oral and dental health is considered an integral part of overall health.

This global burden is further complicated by dental plaque accumulation and the development of dental caries, particularly in children during the preschool and school-age periods. As emphasized by the WHO, this highlights the necessity of instilling lifelong health habits from an early age [23,24]. In this context, the potential contributions of different motivational approaches aimed at increasing the effectiveness of OHE on clinical outcomes were evaluated. However, in this study, it was observed that visual motivational materials with positive or negative content did not provide a statistically significant superiority over standardized one-on-one verbal instructions regarding the clinical parameters examined; accordingly, the H_0_ hypothesis was accepted.

The term “motivation” refers to an individual’s ability to act in accordance with a defined goal and to maintain consistency throughout this process. In the context of education, motivation has been identified as one of the most critical factors in achieving learning objectives [3]. According to Self-Determination Theory, there are two types of motivation: (1) intrinsic and (2) extrinsic. Intrinsic motivation refers to engaging in an activity due to inherent interest, enjoyment, or internal satisfaction perceived by the individual, and it is not applicable to activities lacking such inherent appeal [24,25]. In contrast, extrinsic motivation refers to actions performed under external influences. Both intrinsic and extrinsic motivations can play an important role in maintaining and protecting an individual’s health [26]. In this context, the types of motivation evaluated in the present study can be broadly categorized as extrinsic motivation. The interventions used in the present study—whether visually supported (positive or negative) or based solely on verbal active explanation—aimed to influence behavior through external stimuli rather than the participant’s internal interest. Thus, the visually supported interventions can be classified as approach-based extrinsic motivation (positive visuals) and avoidance-based extrinsic motivation (negative visuals), while the intervention provided to the control groups represents a more neutral form of extrinsic motivation in terms of content.

Considering children’s learning styles and cognitive processing, visual materials are known to be effective in enhancing both learning and motivation [27]. In orthodontic patients, visually demonstrating the consequences of biofilm accumulation has been shown to improve hygiene habits [28]. Although the use of visual materials in OHE is common in the literature, no studies have compared the effectiveness of different types of visual content. In this respect, the present study is a pioneering investigation that objectively examines the impact of visual content differences on motivation.

The delivery of educational modules has been evaluated in the literature using a wide variety of methods and instructor-related factors. The flexibility of OHE lies in its adaptability to various presentation formats, such as traditional classroom teaching, posters, videos, and animations. Moreover, meta-analyses investigating the influence of the educator’s identity have shown that—regardless of whether the presenter is a teacher, dentist, or researcher—the quality of the presentation and the standardization of its content are the key determinants of learning outcomes [29]. In line with this, the educational intervention in the present study was conducted by a researcher (M.İ) who is a PhD candidate in pediatric dentistry and applied a standardized protocol for all participants.

The development of ideal toothbrushing habits requires not only cognitive awareness but also adequately developed motor coordination and hand-eye synchronization [30]. In line with these physiological prerequisites, the age range of 7–14 years was selected in the present study, as it represents a period during which children can both comprehend educational content and perform toothbrushing independently. Additionally, the European Academy of Paediatric Dentistry (EAPD) recommends that children should brush under parental supervision until at least the age of seven; this underscores the post-seven age period as a critical developmental threshold for acquiring independent oral hygiene habits [31]. Given this developmental threshold and the process of acquiring independent habits, the standardization of clinical protocols during the implementation phase is of great importance. Fluoride toothpaste (1000–1450 ppm) and adequate brushing duration are essential in the prevention of dental caries [23,31]. In the present study, to equalize hygiene standards and clearly observe the impact of motivational tools, all participants were advised to brush for 2 min using 1450 ppm fluoride toothpaste and a manual toothbrush [31].

The use of visual materials can serve as engaging and effective short-term stimuli, particularly in children. In pediatric OHE, image cues and interactive educational methods have been reported to be more effective than traditional approaches [6]. For instance, a study demonstrated that the integration of visually interactive game-based support with voice instructions significantly improves knowledge retention and adherence to dental hygiene practices [32]. However, the literature also emphasizes that the effects of such extrinsic stimuli may be limited and insufficient for achieving long-term behavioral change [25,33]. In the present study, whereas the groups utilizing visual aids had a more rapid initial reduction in scores, this difference did not achieve statistical significance when compared to the traditional verbal active education (control groups). In this context, it is evaluated that the clinical improvements observed in all groups over time may be associated with temporary extrinsic regulations rather than permanent intrinsic motivation. However, since behavior changes were monitored for only a one-month period in the present study, it cannot be clearly determined whether these changes transformed into long-term intrinsic motivation. Nevertheless, the gradual decrease in dental plaque scores recorded across all groups over time may indicate a potential trend toward further internalization of behaviors. Nonetheless, due to the one-month follow-up period, it cannot be definitively assessed whether these changes reflect a true transition from extrinsic to intrinsic motivation.

One of the significant findings of the study is that negative visuals, intended to create avoidance motivation, did not yield an additional clinical advantage compared to approach-motivation-based positive visuals or neutral approaches. Although it has been reported in adult literature that the perception of threat can trigger behavioral change [7], this mechanism exhibits developmental and biological differences in children. Indeed, Grima-Murcia et al. demonstrated through electroencephalogram (EEG) findings that the pediatric brain does not prioritize the processing of negative stimuli as much as adults do, and that these stimuli create a limited effect at the behavioral level in children [10]. Additionally, the literature indicates that elements containing high levels of threat may increase anxiety, creating a cognitive load that can lead to a “performance deficit” in the target behavior [11]. Parallel to this biological and psychological framework, Panic et al. emphasized that threat messages regarding oral health in children can only lead to behavioral change when supported by a high sense of self-efficacy [9]. In the present study, the OHE provided to all participants may have neutralized potential anxiety responses or performance declines triggered by negative visuals by supporting the children’s perception of self-efficacy regarding their brushing skills. Consequently, the presence of motivational interviewing and structured education, which are known in the literature to be highly effective in improving oral health behaviors in children, explains why negative stimuli did not offer an additional clinical effect. In other words, once a child acquires a clear method and self-efficacy regarding brushing, an abstract threat stimulus cannot exert a decisive pressure on behavior.

Individual differences may influence motivation, and the effect of extrinsic motivation is believed to vary over time. Indeed, there is strong evidence suggesting that intrinsic motivation leads to more lasting behavioral changes [34]. Similarly, studies in the literature have reported that individuals with intrinsic motivation tend to exhibit more consistent oral hygiene behaviors [24]. From this perspective, the lack of statistically significant differences among the study groups may indicate that extrinsic stimuli alone are not sufficient for sustained motivation in OHE. Therefore, it may be suggested that efforts should focus on strengthening sources of intrinsic motivation.

The selection of time points for outcome measurements in the present study was informed by evidence from previous literature. The 1-week follow-up was chosen to observe the short-term effects of the intervention [21], while the 1-month follow-up was intended to assess its sustained impact over time [35]. Oral health indicators were evaluated at both time points, and the time-dependent effects of the intervention were observed. In all groups, the effect of time was found to be statistically significant in favor of improvement.

Another finding of the study, the observed decrease in halitosis scores running parallel to the reduction in plaque scores, is an expected result. It is well known that the most frequent cause of halitosis is inadequate oral hygiene and the associated microbial activity [36]. In this study, the plaque control achieved—regardless of the type of visual material—appears to have contributed to the improvement in halitosis scores by reducing the bacterial load, which is the source of volatile sulfur compounds.

In the regional evaluation, the more pronounced plaque reduction in anterior regions compared to posterior regions is consistent with data reporting that children tend to focus more on visible/aesthetic surfaces during brushing. Conversely, it is stated that the difficulty of cleaning in posterior regions may be more closely related to performance components such as motor skills and hand-eye coordination [37,38]. These findings suggest that education content should reinforce not only motivational messages but also applied skill development and feedback processes (e.g., tell-show-do, targeted practice) directed toward brushing posterior regions [30].

To enhance sustainability, particularly in posterior region cleaning, it is necessary to go beyond motivational messages and strengthen skill-based practical education components. From a public health perspective, such education protocols that are quickly applicable, low-cost, and standardized hold strategic importance for the scalability of school- or clinic-based programs.

## 5. Limitations

This single-center study provides insights into the effects of different motivational tools used during OHE on children’s oral hygiene performance. Although a single-blind design was implemented for the outcome assessors, the fact that participants and practitioners could not be blinded due to the nature of the educational intervention does not entirely eliminate the possibility of performance bias. However, clinical evaluations were performed by the same individual according to standardized criteria.

Regarding the sample characteristics, excluding post-menarcheal girls—given that hormonal status may influence oral health—reduced biological variability; however, this approach also limited the generalizability of the findings to the overall adolescent female population. Furthermore, the inclusion of a broad age range such as 7–14 years may have led to heterogeneity in how visual motivational tools were perceived and the extent to which they were influential, due to differences in the children’s cognitive and emotional development levels.

In terms of behavioral assessment, the relatively short follow-up period reflects short-term motivational changes rather than long-term oral hygiene habits. While this timeframe is sufficient for observing short-term behavioral changes, longer-term follow-up studies are needed to obtain clearer insights into habit formation and the sustainability of behavioral change.

While the halitosis assessment was performed by a single, well-calibrated examiner, the inherent subjectivity of organoleptic measurement remains a methodological limitation. Additionally, the absence of objective tools to monitor daily oral hygiene and dietary habits required relying on clinical indices for evaluation. The impact of visual materials also warrants further scrutiny, as children’s individual emotional responses were not assessed using standardized psychometric scales. The Hawthorne effect must be considered, as participants’ awareness of being monitored may have influenced short-term clinical improvements.

Additionally, the absence of statistically significant differences between the groups may be attributed to the limited sample size and the potential influence of individual differences in motivation levels.

## 6. Conclusions

The present study demonstrates that the type of visual material is not the primary determinant of short-term clinical improvement. Instead, the key factor is the delivery of OHE through a standardized, structured, and child-centered communicative approach. From a clinical perspective, one-on-one verbal education is an effective core intervention, provided it is supported by adequate duration and feedback mechanisms. In contrast, it is suggested that negative visuals, which were observed to provide no additional clinical benefit, should be evaluated with caution in routine practice due to the potential emotional risks they carry.

In conclusion, the present study demonstrated that both verbal education aided by positive and negative visuals and structured-only verbal education improved children’s oral hygiene and halitosis scores in the short term. Still, the results must be interpreted within the specific context of the study design and the short-term follow-up. Further multicenter, long-term studies with larger sample sizes are needed. Such research will help determine the role of visual tools in the transition to permanent intrinsic motivation and their long-term sustainability.

## Figures and Tables

**Table 1:** The clinical characteristics of the participants.

Variables		n	%
Dentition	Permanent	11	18.3
	Mixed	49	81.7
Dental Malocclusion	Present	34	56.7
	Absent	26	43.3
Anterior Dental Caries	Present	24	40.0
	Absent	36	60.0
**Initial Clinical Assessment**		**Mean**	**Standard Deviation**
Halitosis		1.05	0.99
Total Plaque Score		1.92	0.71
Anterior Plaque Score		1.92	0.79
Posterior Plaque Score		1.89	0.90

**Table 2:** Halitosis Scores for Each Group at Different Time Points.

		Intervention Groups			
	Time	Positive (*n* = 20) (Mean. ± SD)	Negative (*n* = 20) (Mean. ± SD)	Control (*n* = 20) (Mean. ± SD)	*Intervention* × *Time* *Effect*
**Halitosis** **Score**	Baseline	1.20 ± 0.89	1.10 ± 1.11	0.85 ± 0.98	Wilks’ Lambda = 0.930 F(4,112) = 1.028 *p* = 0.396 partial eta square = 0.035
	1st Week	0.90 ± 0.85	0.45 ± 0.75	0.50 ± 0.94	
	1st Month	0.90 ± 0.71	0.35 ± 0.67	0.35 ± 0.58	
	***Time*** ***Effect***	Wilks’ Lambda = 0.655 F(2,56) = 14.766 *p* < 0.001 * partial eta square = 0.345			

* A *p*-value of <0.05 was considered statistically significant.

**Table 3:** Dental Plaque Scores for Each Group at Different Time Points.

			Intervention Groups			
	Region	Time	Positive (*n* = 20) (Mean. ± SD)	Negative (*n* = 20) (Mean. ± SD)	Control (*n* = 20) (Mean. ± SD)	*Intervention* × *Time* *Effect*
**Dental Plaque Score**	**Anterior**	Baseline	1.81 ± 0.80	2.08 ± 0.85	1.86 ± 0.73	Wilks’ Lambda = 0.870 F(4,112) = 2.018 *p* = 0.097 partial eta square = 0.067
		1st Week	1.13 ± 0.58	0.95 ± 0.61	1.15 ± 0.49	
		1st Month	1.14 ± 0.71	0.82 ± 0.44	1.16 ± 0.49	
		***Time*** ***Effect***	Wilks’ Lambda = 0.352 F(2,56) = 51.623 *p* < 0.001 * partial eta square = 0.648			
	**Posterior**	Baseline	1.74 ± 0.69	2.10 ± 1.14	1.81 ± 0.83	Wilks’ Lambda = 0.921 F(4,112) = 1.173 *p* = 0.327 partial eta square = 0.040
		1st Week	1.61 ± 0.86	1.58 ± 1.01	1.49 ± 0.77	
		1st Month	1.53 ± 0.78	1.22 ± 0.65	1.31 ± 0.78	
		***Time*** ***Effect***	Wilks’ Lambda = 0.750 F(2,56) = 9.352 *p* < 0.001 * partial eta square = 0.250			
	**Total**	Baseline	1.80 ± 0.57	2.11 ± 0.89	1.84 ± 0.61	Wilks’ Lambda = 0.870 F(4,112) = 2.021 *p* = 0.096 partial eta square = 0.067
		1st Week	1.41 ± 0.64	1.31 ± 0.75	1.32 ± 0.55	
		1st Month	1.34 ± 0.66	1.04 ± 0.50	1.26 ± 0.54	
		***Time*** ***Effect***	Wilks’ Lambda = 0.446 F(2,56) = 34.789 *p* < 0.001 * partial eta square = 0.554			

* A *p*-value of <0.05 was considered statistically significant.

## Data Availability

The data presented in this study are available on request from the corresponding author. Due to ethical and privacy restrictions, the datasets are not publicly accessible.

## Abbreviations

The following abbreviations are used in this manuscript:

EAPD	European Academy of Paediatric Dentistry
EEG	Electroencephalogram
H	Halitosis
ICDAS	International Caries Detection and Assessment System
MI	Motivational Interviewing
OHE	Oral Hygiene Education
OHRQoL	Oral-Health-Related Quality of Life
PI	Plaque Index
SPSS	Statistical Package for the Social Sciences
WHO	World Health Organization
